# Bio-Engineering of Pre-Vascularized Islet Organoids for the Treatment of Type 1 Diabetes

**DOI:** 10.3389/ti.2021.10214

**Published:** 2022-01-21

**Authors:** Charles-Henri Wassmer, Fanny Lebreton, Kevin Bellofatto, Lisa Perez, David Cottet-Dumoulin, Axel Andres, Domenico Bosco, Thierry Berney, Véronique Othenin-Girard, Begoña Martinez De Tejada, Marie Cohen, Christina Olgasi, Antonia Follenzi, Ekaterine Berishvili, the VANGUARD Consortium, Chiara Borsotti

**Affiliations:** ^1^ Laboratory of Tissue Engineering and Organ Regeneration, Department of Surgery, University of Geneva, Geneva, Switzerland; ^2^ Cell Isolation and Transplantation Center, Department of Surgery, Geneva University Hospitals and University of Geneva, Geneva, Switzerland; ^3^ Faculty Diabetes Center, University of Geneva Medical Center, University of Geneva, Geneva, Switzerland; ^4^ Department of Pediatrics, Gynecology and Obstetrics, Faculty of Medicine, Geneva University Hospitals and University of Geneva, Geneva, Switzerland; ^5^ Department of Health Sciences, University of Piemonte Orientale, Novara, Italy; ^6^ Institute of Medical and Public Health Research, Ilia State University, Tbilisi, Georgia

**Keywords:** regenerative medicine, tissue engineering, β cell replacement therapies, prevascularized iset organoids, human amniotic epithelial cells, HUVECs

## Abstract

Lack of rapid revascularization and inflammatory attacks at the site of transplantation contribute to impaired islet engraftment and suboptimal metabolic control after clinical islet transplantation. In order to overcome these limitations and enhance engraftment and revascularization, we have generated and transplanted pre-vascularized insulin-secreting organoids composed of rat islet cells, human amniotic epithelial cells (hAECs), and human umbilical vein endothelial cells (HUVECs). Our study demonstrates that pre-vascularized islet organoids exhibit enhanced *in vitro* function compared to native islets, and, most importantly, better engraftment and improved vascularization *in vivo* in a murine model. This is mainly due to cross-talk between hAECs, HUVECs and islet cells, mediated by the upregulation of genes promoting angiogenesis (*vegf-a*) and β cell function (*glp-1r*, *pdx1*). The possibility of adding a selected source of endothelial cells for the neo-vascularization of insulin-scereting grafts may also allow implementation of β cell replacement therapies in more favourable transplantation sites than the liver.

## Graphical Abstract

Bio-engineering of pre-vascularized islet organoids for the treatment of type 1 diabetes.
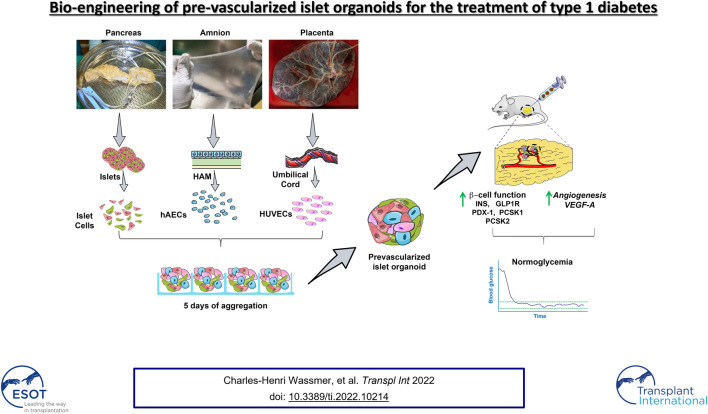

## Introduction

Allogenic transplantation of pancreatic islets is a cell therapy option that holds great promise in the treatment of type 1 diabetes. The development of the Edmonton protocol has drastically increased the success rate of islet transplantation, and has proven to be able to achieve insulin independence in patients with type 1 diabetes (1). Most importantly, pancreatic islet transplantation confers a significant improvement in glycemic control and prevents life-threatening severe hypoglycaemia (2). Despite its efficacy, clinical islet transplantation is facing a number of challenges that limit achievement of steady functional success comparable to whole organ transplantation (3). One of the major challenges is the suboptimal long term graft function caused by the loss of the large portion of intraportally transplanted islets due to the IBMIR reaction, pro-inflammatory microenvironment, low oxygen tension in the liver, impaired vascularization and immunosuppressive drug toxicity (3). Therefore, the search for a suitable alternative transplantation site is a major focus of research in the field. Other limiting factors hampering the widespread application of islet transplantation are shortage of donor organs and need for lifelong immunosuppression (4). Xenogenic islets and stem cell-derived beta cells are the two major potentially unlimited sources of insulin-producing tissue (5).

In recent years, substantial progress has been made in generating and characterizing functional stem cell-derived beta cells, which will undoubtedly change the way we will treat type 1 diabetes (6). The first attempts of clinical application of microencapsulated porcine islets or stem cell-derived endocrine tissue incorporated into macrodevices have already taken place (7, 8) and re-enforce the need to identify a site as functional as portal vein infusion but allowing easy graft removal—a site that to date this remains clinically elusive.

Despite the fact that islets represent only 1–2% of pancreatic tissue volume, they receive 10–15% of the total pancreatic blood flow (9). Each islet possesses 1 to 3 pre-arterioles (10), depending on islet size, that rapidly branch out into a multitude of fenestrated capillaries and form an important intra-islet micro-circulation that is five time denser than in the exocrine tissue (11). The cross-talk between endocrine and endothelial cells is vital for proper islet development, configuration and vascularization. Islet cells secrete vascular endothelial growth factor-A (VEGF-A) and angiopoietin-1 in order to recruit endothelial cells (ECs) that are necessary for islet development, survival and function. On the other hand, ECs are involved in cell differentiation, insulin gene expression and cell segregation during embryogenesis (12, 13). In addition, they secrete components of the intra-islet basement membrane that are crucial for proper endocrine function (11).

Islet isolation and culture lead to the disruption of the islet capillary system, with significant loss of ECs due to de-differentiation or necrosis (14). In addition, islets vary in size, ranging from 50 to 400 μm in diameter. In the immediate post-transplantation period, avascular islets are supplied with oxygen and nutrients solely by diffusion until re-establishment of the blood flow, a process that can take about 2 weeks (9). Because of that, larger islets fail to engraft due to insufficient vascularization and subsequent necrosis (15). Significant efforts have been made to develop new strategies to minimize hypoxia-induced β cell death.

Several scientific groups, including our own, have demonstrated that re-aggregation of islet cells in combination with other cell types into homogeneous, round shaped and size-controlled spheroids leads to improvement of function and viability, thanks to heterotypic cell–cell interactions and reproduction of the complex natural morphology of the islet (16–20). In our previous studies, we have shown that incorporation of human amniotic epithelial cells (hAECs) into insulin-secreting organoids protected islet cells from oxidative stress *in vitro*, subsequently improving ß cell viability, function and engraftment (17, 20). Here, we propose an improved approach, in which we engineer pre-vascularized organoids that provide both control over their size and composition, and prompt re-establishment of the cross-talk between ECs and islet cells, thereby facilitating graft revascularization after transplantation.

## Materials and Methods

### Reagents and Antibodies

All reagents and antibodies used in this study are listed in Supplementary Tables S1–S3.

### Animals

Animal experiments were performed in accordance with the Geneva veterinary authorities and approved by the Institutional Animal Care and Use Committee of the University of Geneva. Ten-week-old, pregnant female, Lewis rats were purchased from Janvier Laboratory (Le Genest St-Isle, France) and bred in our animal facility at the Geneva University. Fifteen-to 21-week-old male rats were used for pancreatic islet isolation. Six-to 9-week old male B6.129S7‐Rag1^tm1Mom^/J (abbreviated NOD–*Rag1* ^*null*^ bred at Charles River Laboratories, Saint‐Germain‐Nuelles, France) mice were used as transplantation recipients. All animals were kept under conventional housing conditions with free access to water and food.

### Human Tissues

Studies involving human tissues were approved by the Commission Cantonale d’Ethique de la Recherche (CCER; protocol PB_2017-00101), in compliance with the Swiss Human Research Act (810.30).

Placentas were obtained from women undergoing elective caesarean section of uncomplicated, term pregnancies. Informed, written consent was obtained from each donor prior to tissue collection.

### Isolation and Culture of Human Umbilical Vein Endothelial Cells and Human Amniotic Epithelial Cells

Human umbilical vein endothelial cells (HUVECs) were isolated using a method adapted from a previously published protocol (21). Briefly, the umbilical vein was rinsed, then distended with Collagenase A solution (2 mg/ml) and incubated at 37°C for 12 min. Released cells were then collected by flushing the vein with cold HBSS supplemented with 10% fetal bovine serum (FBS), 100 U/ml penicillin, 100 mg/ml streptomycin and 0.25 mg/ml amphotericin B. Isolated HUVECs were plated in a 75 cm^2^ flasks and cultured at 37°C, 21% O_2_ and 5% CO_2_ in M199 medium supplemented with 20% FBS, 100 U/ml Penicillin and 0.1 mg/ml Streptomycin (1% of a L-Glutamin-Penicillin-Streptomycin stock solution), Fungin 0.1%, 30 μg/ml endothelial cell growth supplement and 100 μg/ml heparin. HUVECs from passage 2 to 7 were used in this study.

hAECs were isolated, cultured and characterized as described previously (10, 14). Freshly isolated hAECs were cultured in DMEM/F-12 medium, supplemented with 10% FBS, 2 mmol/l L-Glutamin, 100 U/ml Penicillin, and 0.1 mg/ml Streptomycin (1% of a L-Glutamin-Penicillin-Streptomycin stock solution, 1 mmol/L sodium pyruvate, 1% MEM NEAA 100X, 0.1% fungin, 0.05 mmol/L 2-mercaptoethanol, 10 ng/ml human recombinant epidermal growth factor (EGF). Only cells at passage 1 were used in this study.

Medium was changed every 48 h. Confluent cells were recovered by mild trypsinization and were cryopreserved for later utilization.

### Rat Islet Isolation and Dissociation

Rat islets were isolated by enzymatic digestion (collagenase V) and purified using a discontinuous Ficoll gradient (22–24). Isolated islets were cultured (37°C, 5% CO_2_) in DMEM medium supplemented with 10% FBS, 2 mmol/L L-glutamine, 100 U/ml penicillin, 0.1 mg/ml 1 mmol/L sodium pyruvate and 11 mmol/L glucose for 24 h. Islets were then dispersed into single islet cells (ICs) by incubation in 0.05% trypsin-EDTA (16).

### Characterization of Human Umbilical Vein Endothelial Cells and Human Amniotic Epithelial Cells

HUVECs and hAECs were analyzed for expression of previously reported endothelial cell surface markers or specific amniotic epithelial cell surface markers by flow cytometry.

For analysis, cells (2.5 × 10^5^) were stained by incubation for 30 min with primary or isotype control antibody in 100 µl PBS with 0.2% BSA, washed twice with PBS, and analyzed. Antibodies used for HUVECs were: AlexaFluor 657-conjugated anti-CD144 (1:40 dilution), PE-conjugated anti-CD31 and PerCP-Cy 5.5-conjugated anti-CD45 (1:25 dilution). Antibodies used for hAECs were: FITC-conjugated anti-human CD105 (clone 266), BV421-conjugated anti-human CD326 (clone EBA-1), PerCP-Cy5.5 conjugated anti-SSEA4 (clone MC813-70) (1:50 dilution), PE-Cy7 conjugated anti-human CD90 (clone 5E10; 1:100 dilution), PE-conjugated anti-human HLA-E (clone 3D12) and APC-conjugated anti-human HLA-G (clone 87G; 1:20 dilution).

Flow cytometry analysis was performed on a Gallios cytometer using the Kaluza Analysis software.

HUVECs were further characterized by immunostaining. Immunofluorescent assessment was performed on the cells cultured on gelatine-coated glass coverslips. Fixed cells were washed, permeabilized and stained with the following primary antibodies: mouse anti-CD31 (1:50 dilution), rabbit anti-von Willebrand factor (1:100 dilution) and mouse anti-vimentin (1:50 dilution). Cells were then incubated with corresponding Alexa Fluor and FITC-conjugated secondary antibodies. For nuclear counterstaining samples were mounted with aqueous solution containing 4,6 diamidino-2-phenylindole (DAPI).

### Functional Assessment of Human Umbilical Vein Endothelial Cells *In Vitro*: Tube Formation Assay

The tube formation assay was performed according to manufacturer’s protocols of Corning® Matrigel® Matrix. Briefly, Matrigel thawed overnight at 4°C was mixed with VEGF (200 ng/ml) and 250 μl of matrix was added to each well osf 24-well plates. After 1 h of incubation at 37°C, cells (8 × 10^4^) were seeded onto the Matrigel and tube formation of HUVECs was observed and photographed using an inverted phase-contrast microscope during 6 h.

### Lentiviral Transduction

Lentiviral vector carrying the green fluorescent protein (GFP) under the control of an endothelial specific promoter Vascular endothelial cadherin (VEC/Cdh5) (LV-VEC.GFP) was provided by Prof. A. Follenzi (Università del Piemonte Orientale). HUVECs were transduced with LV-VEC.GFP at passage 3 using a multiplicity of infection (MOI) of 10 (MOI = 10). Transduction efficiency was assessed by fluorescent microscopy and flow cytometry and considered successful when at least 80% of cells showed expression of GFP.

### Generation of Pre-Vascularized Islet Organoids

Pre-vascularized islet organoids (PIO) were generated on AggreWell™400 24-well plates by seeding mixture of ICs, HUVECs and hAECs at a ratio of 5:4:1 (800 cells/organoid). Undissociated native islets (NI), ICs spheroids (400 ICs/spheroid), hereafter referred to as pseudo-islets (PI), and IC:HUVEC spheroids (ratio 1:1, 800 cells/spheroid), hereafter referred to as IC + HUVEC served as controls. PIO, PI and IC + HUVEC were cultured for 4 days to allow cell aggregation at 37°C, 21% O_2_ and 5% CO_2_.

Culture medium for PIO was prepared by mixing equal volumes of complete DMEM, DMEM/F12 and M199 medium, hereafter referred to as organoid medium. IC + HUVEC were cultured in the mixture of complete DMEM and M199 medium at the ratio 1:1. Finally, PI and NI were cultured in complete DMEM medium. Culture medium was changed every other day. Mean diameter of NI, PIO and PI were calculated on the images taken on light microscope using ImageJ software.

In order to observe PIO composition and cell distribution during culture, fluorescent carbocyanine dyes CM-DiL (red) prelabeled hAECs and GFP transduced HUVECs were used. Pictures were taken using an epifluorescent microscope (DMi8 manual microscope).

PIO, PI and NI were collected fixed in formalin and embedded in paraffin. Serial sections of 5 μm were cut and processed for immunofluorescent staining. Slides were stained with the following primary antibodies: guinea pig anti-insulin (1:100), chicken anti-GFP (1:500), and rabbit anti-CK-7 (1:100). The following secondary antibodies were then applied: donkey anti–guinea pig Alexa 555 Fluor-conjugated (1:300), donkey anti–guinea pig FITC‐conjugated (1:200), donkey anti-mouse AMCA-conjugated (1:50), goat anti-chicken Alexa Fluor 488 (1:500).

### Organoids Sprouting Assay

One hundred PIO were resuspended in a collagen solution, transferred into prewarmed 24‐well plates and allowed to gelify for 30 min. Next, 0.1 ml organoid medium supplemented with VEGF-A at the concentration of 200 ng/ml was pipetted on top of each hydrogel containing PIO. The hydrogels were cultured for 24 h at 37°C, 5% CO_2_, and 100% humidity. As control, one hundred IC + HUVEC spheroids and PI were cultured in the same way in the hydrogel.

### *In Vitro* Functional Assessment

To assess functional capacity, 300 NI and an equivalent number of PIO and PI, were incubated in duplicates for 1 h at 37°C in Krebs–Ringer solution containing low glucose (2.8 mmol/L) in order to equilibrate the samples. After a change of medium, islets and aggregates were incubated at 37°C for another hour in Krebs–Ringer solution containing low glucose (2.8 mmol/L), followed by 1 h at high glucose (16.7 mmol/L). Supernatants were collected and stored at −20°C. Insulin concentration in supernatants was measured using a rat insulin ELISA kit and normalized to the total insulin content. Results are expressed as the ratio between insulin secreted in high glucose to low glucose, referred to as stimulation index (SI). In addition, total insulin content per IC was measured by dividing the total insulin content by the number of ICs present in the NI, PI and PIO.

### Diabetes Induction and Xenogeneic Transplantation

Three days before transplantation mice were subjected to intraperitoneal injection of STZ (180 mg/kg). Non-fasting blood glucose levels were then checked daily using a portable glucometer. Only mice with blood glucose levels over 18 mmol/L for 3 consecutive days were used in this study. Glycemia readings over 28 mmol/L, indicated as “high” on glucometer, were recorded as 30 mmol/L.

A marginal mass of 300 islet equivalents (IEQ) for NI and 1200 PIO, PI and IC + HUVEC were transplanted. Number of organoids was based on the average number of islet cells per IEQ, previously estimated as 1,560 ICs/IEQ (25).

At the day of transplantation, NI and engineered constructs were recovered from culture, packed in PE50 tubing and transplanted into the epididymal fat pad (EFP) of diabetic mice. Non-fasting glucose was assessed daily during the first week and 3 times per week thereafter. Normoglycemia was defined as two consecutive blood glucose levels under 11.1 mmol/L.

### Graft Metabolic Function Assessment

Graft capacity to clear glucose *in vivo* was assessed dynamically by intraperitoneal glucose tolerance test (IPGTT) at 30 days after transplantation. Mice were fasted for 6 h and intraperitoneally injected with 2 g of glucose/kg. Blood glucose measurements were taken at 0, 15, 30, 45, 60 and 120 min.

### Lectin Injection

Functional graft vasculature was assessed by infusing DyLight 594-conjugated Lycopersicon Esculentum (Tomato) lectin into the beating left ventricle of mice hearts. Mice were injected with 100 μl of undiluted lectin. Lectin was allowed to circulate for 1 min. Then, the right ventricle was cut to allow blood flow decompression and a volume of 3 ml of PBS was injected into the left ventricle, followed by 1 ml of 4% PFA. The graft bearing EFPs were collected and fixed overnight in 4% PFA at 4°C. They were then maintained in 30% sucrose at 4°C until used for histology.

### Immunohistological Assessment of Recovered Grafts

Grafts were recovered, fixed in formalin and embedded in paraffin. Serial sections of 5 μm were cut and processed for immunofluorescent staining. Tissue samples were permeabilized with 0.5% Triton X-100/PBS for 30 min, followed by 1-h incubation in 0.5% BSA/PBS at room temperature to block unspecific sites. Slides were then incubated with the following primary antibodies: guinea pig anti-insulin (1:100), rabbit anti-CD34 (1:2,000), chicken anti-GFP (1:500), and rabbit anti-VEGF (1:100). The following secondary antibodies were then applied: donkey anti–guinea pig Alexa 555 Fluor-conjugated (1:300), donkey anti–guinea pig FITC‐conjugated (1:200), donkey anti‐rabbit Alexa 555 Fluor-conjugated (1:300) and goat anti-chicken Alexa Fluor 488 (1:500). Both primary and secondary antibodies were diluted in PBS-0.5% BSA. Finally, slides were mounted with aqueous mounting medium containing DAPI for nuclear staining. Slides were processed on a Zeiss Axioscan.Z1 slide scanner and a Zeiss Axiocam. To analyse vascularization, six pictures per condition were taken and the number of CD34^+^ cells were counted and normalized by the graft area.

Morphometric analysis was performed using Zen 2.3 Blue Edition software.

### Real-Time Quantitative PCR

Graft bearing EFPs recovered at 3 and 30 days after transplantation were processed for PCR analysis. RNA was extracted using the RNeasy minikit and reverse transcribed with a High Capacity cDNA Reverse transcription kit. Gene amplification was performed by RT-PCR using TaqMan Fast Advance Master Mix. Primers used for amplification are listed in Supplementary Table S4. *RPLP1* was used as a housekeeping gene to normalize gene expression values. Data were calculated using the comparative cycle threshold Ct method (2^−ΔCt^ method) and are expressed in arbitrary units.

### Statistical Analysis

Continuous variables are expressed as mean ± SEM. Multiple comparisons were analyzed using one-way ANOVA followed by Dunnett multiple comparisons test while two-way comparisons were analyzed using the Student’s t-test. Cumulative number of animals reaching normoglycemia was compared using the log‐rank (Mantel‐Cox) test. A *p* value ≤0.05 was considered statistically significant. All statistical analyses were performed with the Prism software 8.0.

## Results

### Human Umbilical Vein Endothelial Cell Characterization and Transduction

**FIGURE 1:**
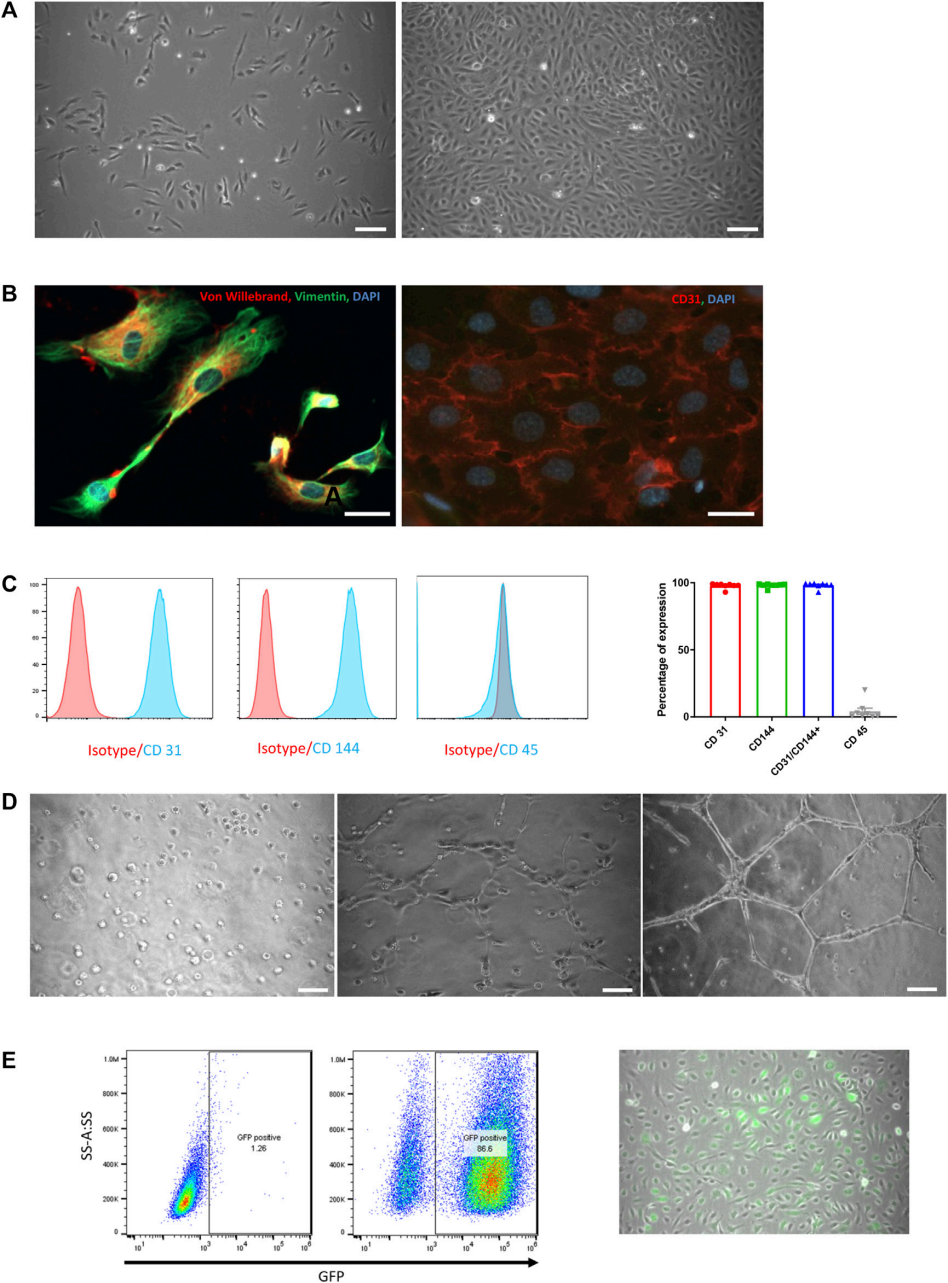
HUVEC characterization and *in vitro* functional assessment. **(A)** Phase-contrast microscopic pictures of HUVEC in culture at day 1 and day 5. Scale bar = 50 µm. **(B)** Immunofluorescence staining of cultured HUVEC with von Willebrand (red) and Vimentin (green, left panel) and CD31 (red, **right panel)**. Nuclei are labelled with DAPI (blue). Scale bar = 25 µm. **(C)** FACS analysis on HUVEC for CD31, CD144 and CD45 with their respective isotypes **(left panels)** and expressed as the percentage of positivity of expression on 8 consecutive preparations (mean ± SEM, **right panel**). **(D)** Phase-contrast microscopic pictures of tube formation assessment on Matrigel at 0 h, 2 and 6 h. Scale bar = 50 µm. **(E)** Assessment of GFP transduction success by flow cytometry analysis **(left panel)** and by phase-contrast microscopic images (right panel). GFP-positive cells are spontaneously green, scale bar = 50 µm.

HUVECs reached 80% confluence within 5 days with initial seeding density of 6,000 cells/cm^2^. Morphologically, cells displayed typical elliptic shape (Figure 1A) and were positive for von Willebrand factor and CD31 (Figure 1B). Endothelial origin of the cells was additionally confirmed by flow cytometry. Cells were positive for CD31 and CD144 (97.8% ± 0.7 and 98.1% ± 0.6, respectively) and negative for CD45 (95.8%) (Figure 1C).

When cultured on Matrigel, HUVECs formed well-shaped vascular-like structures over a period of 6 h (Figure 1D).

To track HUVECs within organoids both *in vitro* and *in vivo*, cells were transduced with LVs carrying green fluorescent protein (GFP) gene under the control of the VEC promotor. HUVEC positivity for GFP was observed during culture and confirmed by flow cytometry 3 days after transduction with 86.6% of GFP+ cells (Figure 1E right and left panel, respectively).

### Human Amniotic Epithelial Cells Characterization

hAECs used in this study were isolated from six different placentas. Flow cytometry analysis demonstrated strong positivity of hAECs for the embryonic cell surface marker SSEA-4 (88.4 ± 5.0%) and the epithelial cell adhesion molecule (CD326; 95.9 ± 1.3%). HLA-E and HLA-G were expressed in 16.9 ± 4.7% and 48.6 ± 12.3% of the cells, respectively. Finally, expression of CD105 and CD90 by hAECs were 17.6 ± 5.6%, 50.1 ± 7.1, respectively. The results of each hAEC preparation are described in Supplementary Figure S1.

### Cellular Composition, Endocrine Function and Angiogenic Activity of Pre-Vascularized Islet Organoids

**FIGURE 2:**
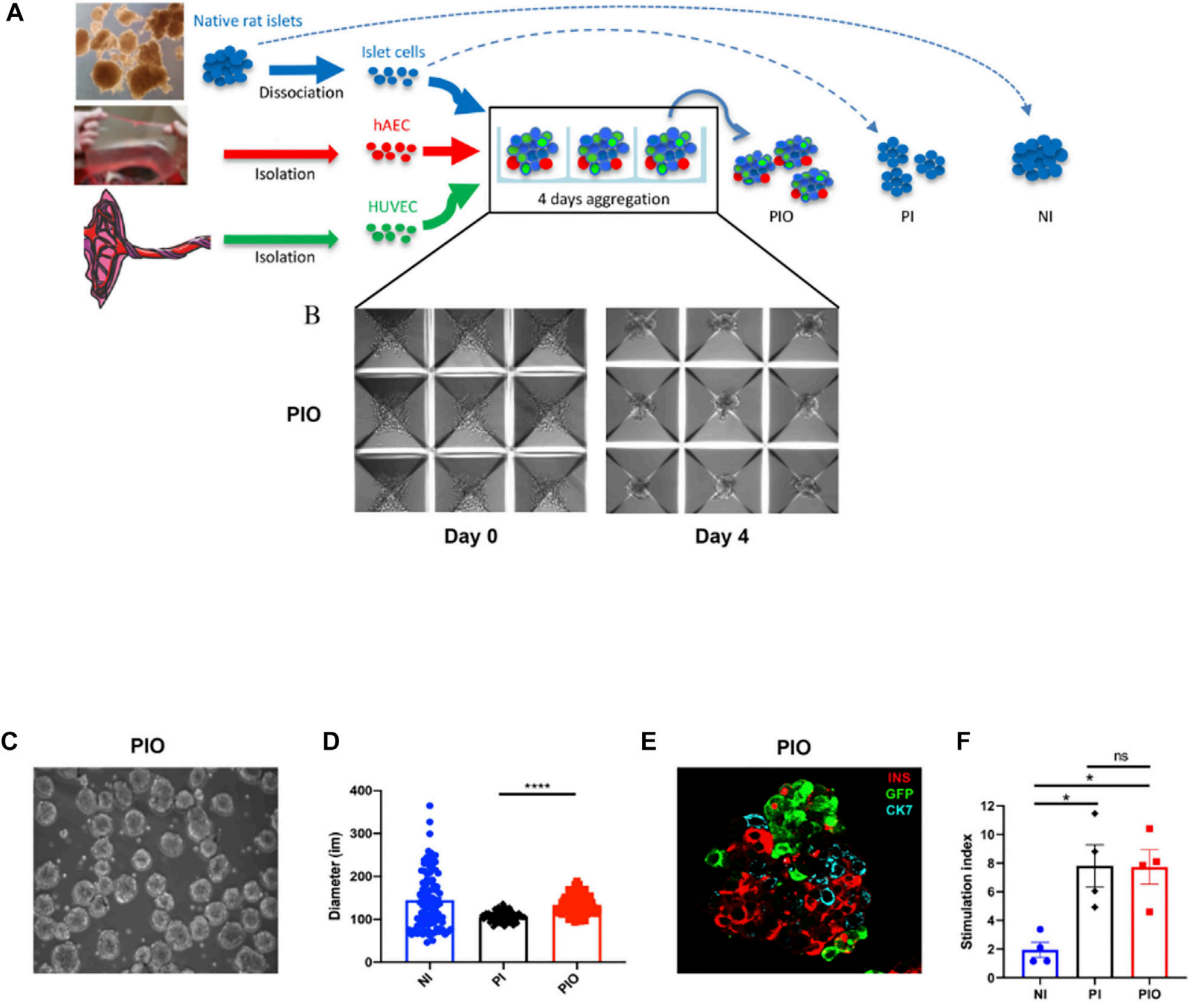
Organoids generation. **(A)** Schematic representation of PI and PIO generation in culture. **(B)** Light microscope pictures of the PIO cultured in AggreWell™400 24-well plates at day 0 and day 4. Scale bar = 100 µm. **(C)** Light microscope pictures of the PIO after collection from the wells. **(D)** Average diameter of each condition calculated at 4 days of culture (*n* = 100/condition). **(E)** Representative immunofluorescence stainings of PIO. Islet cells are stained for insulin (red), HUVECs for GFP (green) and hAECs for CK7 (blue). Scale bar = 25 µm. **(F)** *In vitro* function assessed by GSIS and represented by the stimulation index (*n* = 4). All data are expressed as mean ± SEM. *p < 0.01, ***p < 0.001, one-way ANOVA with Dunnett’s multiple comparison test.

Generation of PIO and PI is described in Figure 2A. Aggregation and incorporation of the different cell types occurred within 4 days (Figures 2B,C). Mean diameter of NI, PI and PIO was 144.4 ± 6.6, 105.8 ± 1.2 and 134.3 ± 2.3 μm, respectively (Figure 2D). NI showed the biggest heterogeneity in size. PI exhibited a significantly smaller mean diameter in comparison with PIO (*p* < 0.0001), due to fewer cellular content. Cellular composition observed by fluorescent microscopy showed that all 3 cell types were present in the PIO (Figure 2E). The functional capacity of the constructs was evaluated by glucose-stimulated insulin secretion (GSIS) assay. PI and PIO demonstrated significantly improved insulin secretion in response of glucose stimulation (SI = 7.8 ± 1.5 and 7.7 ± 1.2), compared to NI (SI = 2.0 ± 0.5, *p* = 0.013 and *p* = 0.014, respectively). No significant difference was observed between PI and PIO (Figure 2F). In addition, total insulin content/IC was measured and compared between the three groups. PI and PIO demonstrated an increased insulin content/IC (0.01 ± 0.003 and 0.008 ± 0.002 pmol/L, respectively) in comparison with NI (0.002 ± 0.0004 pmol/L). These dramatic enhancement of static GSIS secretion in our constructs compared to unmodified native islets indicate that better oxygen and nutrient access, and improved transport of glucose and insulin, enhanced survival and function of PI and PIO. Our findings are consistent with previous reports on better *in vitro* performance of smaller pseudoislets (26, 27).

To investigate the angiogenic potential of the PIO, collagen-based sprouting assays were performed. Our results demonstrated that PIO showed more extensive sprouting in surrounding matrix compared to IC + HUVEC (Supplementary Figure S2). In contrast, no sprouting was observed from PI (data not shown). Furthermore, immunofluorescence revealed GFP positive cells confirming their endothelial nature.

### Pre-Vascularized Islet Organoids Improve Glycaemic Control in Immunodeficient Diabetic Mice

**FIGURE 3:**
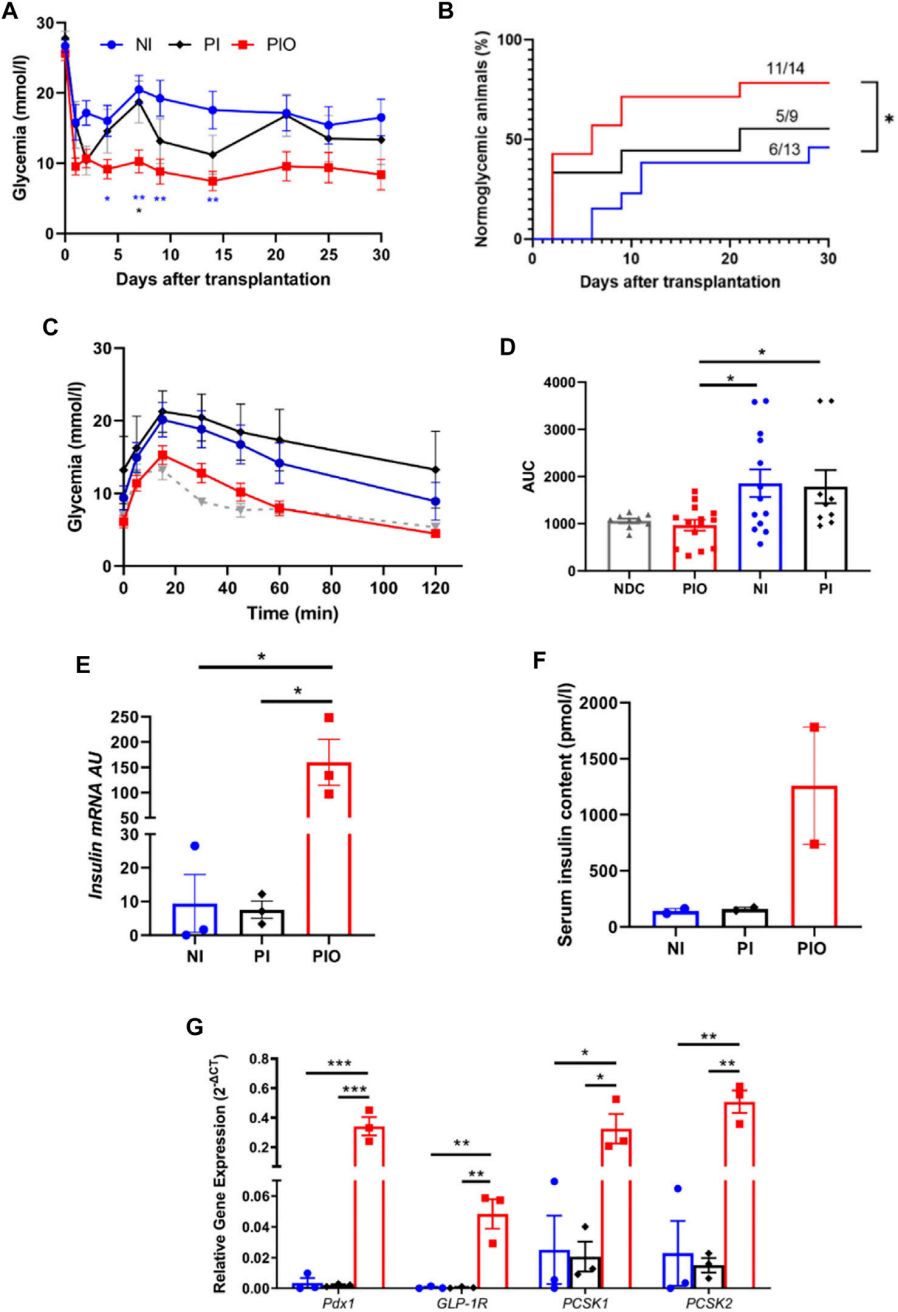
*In vivo* function of organoids in immunodeficient, diabetic mice. **(A)** Glycemia level measured over 30 days in NOD‐*Rag1* ^*null*^ mice transplanted with 300 NI (*n* = 13, blue circle) and their equivalent number of PI (*n* = 9, black diamond) and PIO (*n* = 14, red square). Mean glucose level was compared at 4, 7, 9, 14, 21 and 30 days by a one-way ANOVA with Dunnett’s multiple comparison test. All data are expressed as mean ± SEM. *p < 0.05, **p < 0.01. **(B)** Cumulative number of mice reaching normoglycemia over 30 days. Comparison made using the log‐rank (Mantel‐Cox) test, *p < 0.05. **(C–D)** Glycemia level of each group during the intraperitoneal glucose tolerance test performed at 30 days post-transplantation **(C)** and their corresponding AUC values **(D)**. Grey triangles represent the non-diabetic control (NDC) group (*n* = 9). **(E)** Insulin mRNA expressed by NI, PI and PIO at 30 days post-transplantation; insulin mRNA was analyzed by qPCR, arbitrary units (AU) after normalization to housekeeping genes. Data shown are mean ± SEM, *p < 0.05, one-way ANOVA with Dunnett’s multiple comparison test, *n* = 3. **(F)** Insulin concentration measured by ELISA in mice serum at 30 days post-transplantation. All data are expressed as mean ± SEM, one-way ANOVA with Dunnett’s multiple comparison test, *n* = 2. **(G)** *pdx1, glp-1r, pcsk* and *pcsk2* expressed in PIO (red columns), PI (black columns) and NI (blue columns) at 30 days after transplantation, data presented as arbitrary units (AU) after normalization to housekeeping genes. Data shown are means ± SEM. *p < 0.05, **p < 0.01, ***p < 0.001 and comparisons were made by a one-way ANOVA with Dunnett’s multiple comparison test, *n* = 3.

To assess whether incorporation of hAECs and HUVECs into the islet organoids could promote engraftment and function *in vivo*, diabetic NOD–*Rag1* ^*null*^ mice were transplanted with a marginal mass of PIO (*n* = 14), NI (*n* = 13) and PI (*n* = 9). Mice transplanted with PIO demonstrated significant improvement of glycaemic control compared to both controls. Average blood glucose levels were significantly lower in the PIO group compared to NI and PI (Figure 3A). Normoglycemia was reached in 78.6% of animals (11/14) in the PIO group, in comparison with 55.6% (5/9) and 46.2% (6/13) for the PI and NI groups, respectively (Figure 3B). Median time to achieve normoglycemia was 6 days in the PIO group, 21 days in the PI group and >30 days in the NI group. To investigate secretory function of the graft, IPGTT was performed at 30 days post-transplantation. Mice transplanted with PIO and non-diabetic controls (NDC) showed lower blood glucose levels when compared to animals transplanted with PI and NI (Figure 3C). This is illustrated by the increasing area under the curve (AUC) of the different groups, with PIO (966.8 ± 113.7), PI (1783 ± 351.1, *p* = 0.05 vs. PIO) and NI (1856 ± 294.5, *p* = 0.014 vs. PIO; Figure 3D).

We further investigated whether the improved glycemic control in the PIO group was associated with insulin production from the transplanted β cells. Remarkable upregulation of rat insulin mRNA levels in the graft was found in the PIO group in comparison to controls (PIO vs. PI, *p* = 0.013, PIO vs. NI, *p* = 0.013; Figure 3E). These results were supported by insulin measurements in the serum taken from the same mice (Figure 3F). Although a statistical significance wasn’t achieved, a ten-fold increase in insulin levels was detected in the PIO group (1,259 ± 521 pmol/L), in comparison to both controls (NI: 140.6 ± 22.1 pmol/L, PI: 159.8 ± 14.4 pmol/L, *p* = ns).

*Glp-1r*, *pdx1* are known to be critical for promoting insulin secretion (28–31). Therefore, we investigated whether these genes were involved in the improved secretory outcomes of PIO. Gene expression analyses revealed upregulation of genes involved in β-cell function (*pdx1*, *pcsk1*, *pcsk2* and *glp-1r*) in PIO at 30 days post-transplantation, compared to controls (*pdx1*: PIO vs. PI, *p* = 0.0009, PIO vs. native islet, *p* = 0.0009; *glp-1r*: PIO vs. PI, *p* = 0.002, PIO vs. native islet, *p* = 0.002; *pcsk1*: PIO vs. PI, *p* = 0.02, PIO vs native islet *p* = 0.021 and *pcsk2*: PIO vs. PI, *p* = 0.0005, PIO vs. native islet, *p* = 0.0006; Figure 3G). Interestingly, at an earlier time points (3 days), a similar increase in gene expression was observed in PI and PIO in comparison with NI group, although without reaching statistical differences (Supplementary Figure S3). These data indicate that incorporation of accessory cells into the organoids supports long term secretory function of β cells.

### Transplantation of Pre-Vascularized Islet Organoids Accelerates Graft Revascularization

**FIGURE 4:**
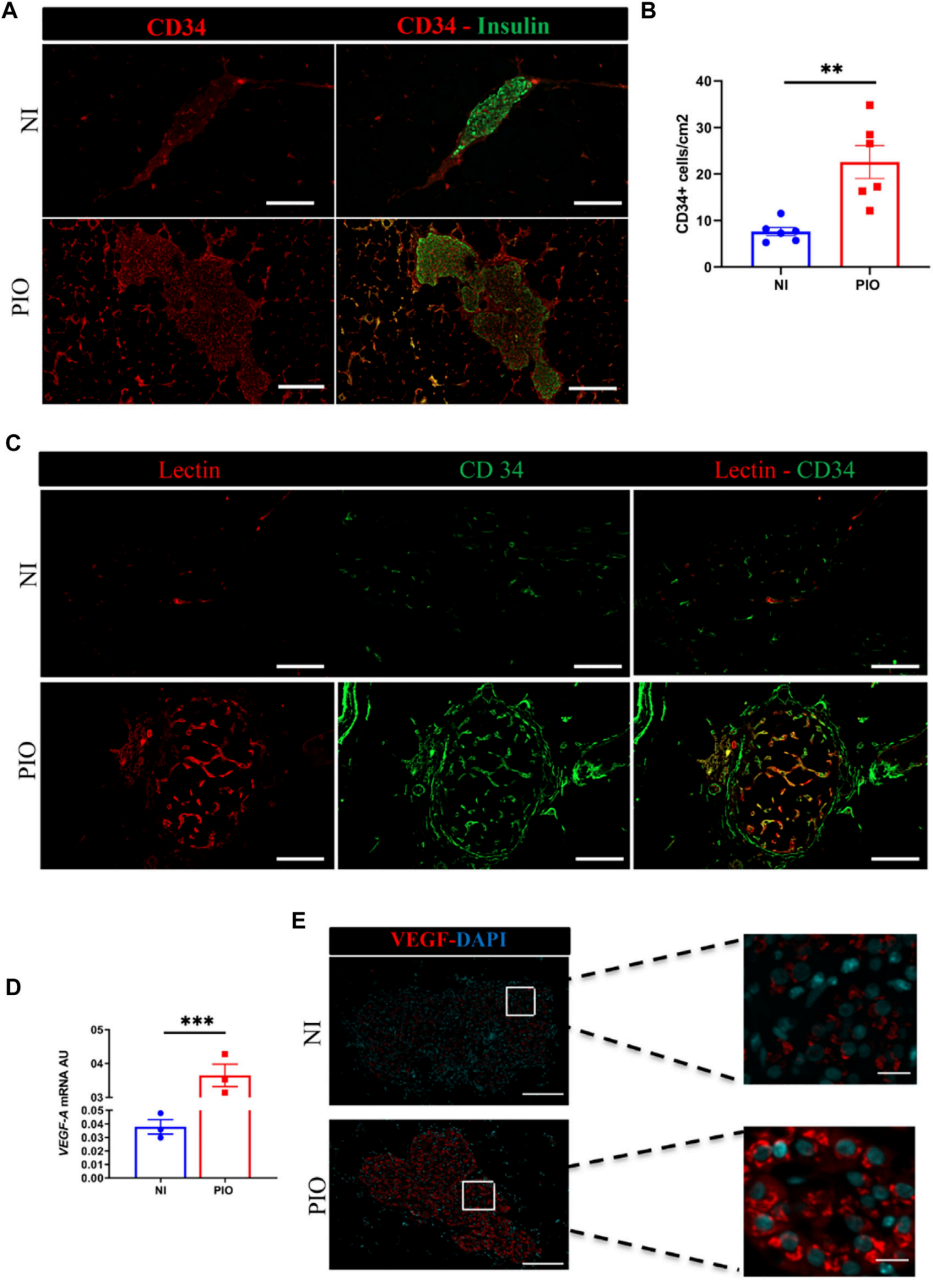
*In vivo* revascularization assessment by immunohistological analysis. **(A)** The blood vessels of the graft detected at day 30 post-transplantation using CD34 (red) and insulin (green) immunostaining. Grafts Scale bar = 50 µm. **(B)** Quantitative analysis of revascularization was achieved by calculating the number of CD34 positive cells in the insulin positive area and the result was divided by the graft surface area. This was realized in two graft regions per mouse and in 3 mice per group. All data are expressed as mean ± SEM. *p < 0.05, **p < 0.01, comparisons were made by a 2-tail unpaired Student *t* test. **(C)** Assessment of vessel functional capacity by mice injection of 100 µl of lectin. Capillaries are labelled in red and endothelial CD34+ cells in green. Scale bar = 50 µm. **(D)** *vegf-a* mRNA expression analyzed by qPCR at 30-days post-transplantation in PIO and NI groups; data presented as arbitrary units (AU) after normalization to housekeeping genes. Data shown are expressed as mean ± SEM. ***p < 0.0006, 2-tail unpaired Student t test, *n* = 3. **(E)** Recovered grafts stained for VEGF-A at day 30 after transplantation. Scale bars = 100 μm.

To evaluate engraftment and revascularization, graft-bearing EFPs were removed at 30 days post-transplantation and processed for histology. Immunohistochemical staining for CD34, a marker for endothelial cells, showed that vessel density was significantly higher in the PIO samples (22.6 ± 3.5 CD34 + cells/cm^2^) than in the NI samples (7.6 ± 0.9, *p* = 0.002; Figures 4A,B). Furthermore, in the PIO group, vessels were observed not only around graft, but mainly within β-cell positive area.

To investigate whether the blood vessels formed within the engrafted tissue constructs become functional and contribute to graft perfusion, we used intravascular injection of fluorescently labeled Lectin. Histological assessment of the Lectin-perfused grafts demonstrated the presence of functional Lectin positive vascular network within the PIO, in contrast only few vessels were present within NI (Figure 4C).

Next, we examined the mechanisms by which supportive cells (HUVECs and hAECs) contributed to rapid neovascularization of the graft. To this end, we investigated whether these cells might induce the production of angiogenic factors, such as *vegf-a* (Figure 4D). We observed, that rat *vegf-a* mRNA expression was significantly higher in PIO group (0.365 ± 0.033 AU) compared to NI (0.038 ± 0.005 AU; *p* = 0.0006) group. This finding was further confirmed by immunohistochemical staining for *vegf-a* of recovered samples, demonstrating higher fluorescent intensity in the PIO compared to NI (Figure 4E). These data indicate that incorporation of HUVEC and hAEC into PIO contribute to graft revascularization.

### Human Amniotic Epithelial Cells Incorporation Into Organoids Improves Function and HUVEC-Derived Revascularization

Finally, we evaluated whether incorporation of hAECs into the organoids was essential for the engraftment and vascularization of the PIO. To this end, we added an additional group of mice transplanted with spheroids composed of IC: HUVEC (1:1 ratio) to the three existing groups.

**FIGURE 5:**
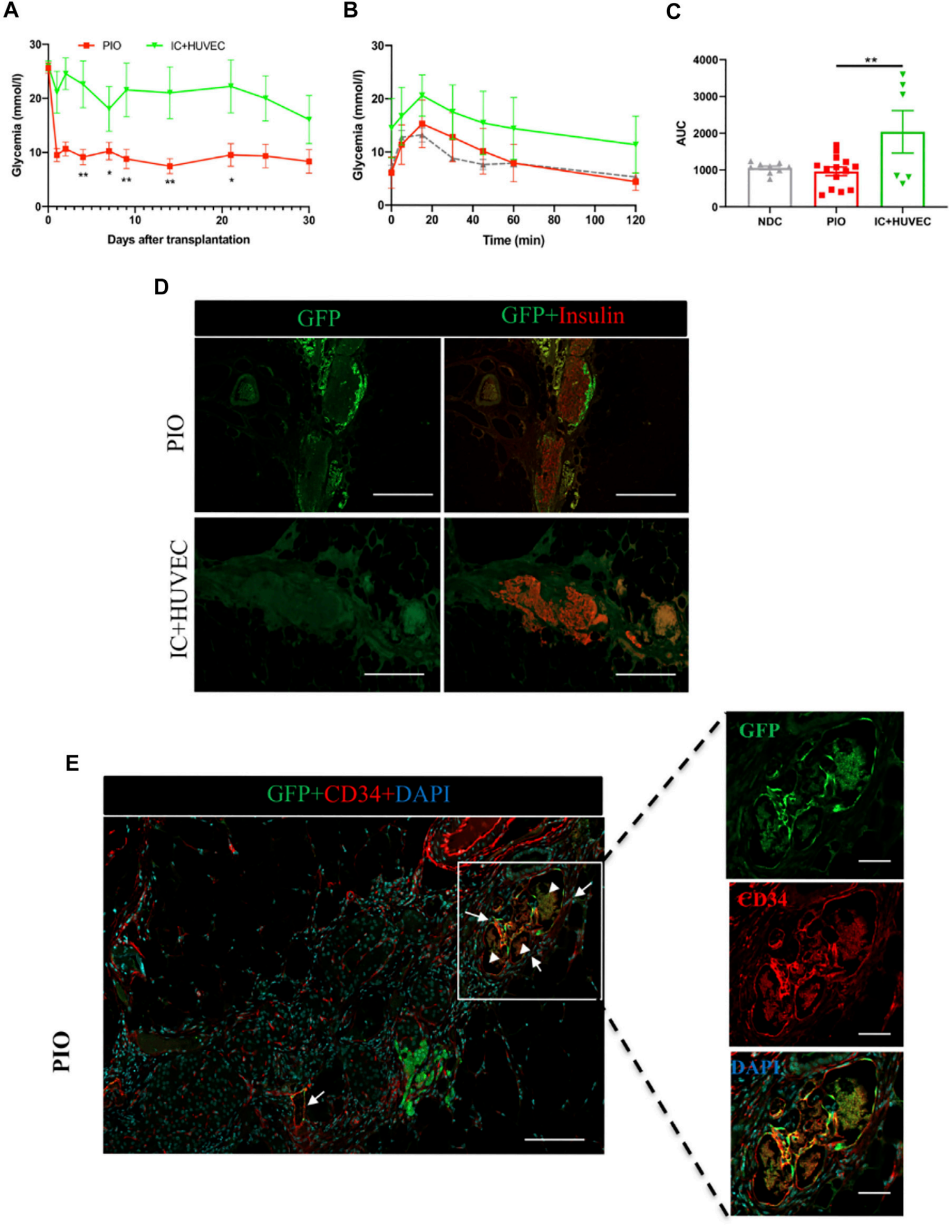
*In vivo* function of IC + HUVEC spheroids, in immunodeficient, diabetic mice. **(A)** Mean glucose levels measured in NOD‐*Rag1* ^*null*^ mice transplanted with PIO (*n* = 14, red squares) and IC + HUVEC (*n* = 6, green inverted triangles). Mean glucose level was compared at 4, 7, 9, 14, 21 and 30 days post-transplantation by a 2-tail unpaired Student *t* test. All data are expressed as mean ± SEM. *p < 0.05, **p < 0.01. **(B,C)** Intraperitoneal glucose tolerance test performed at 30 days post-transplantation and their corresponding AUC. Grey triangle represents the non-diabetic control (NDC) group (*n* = 9). Comparisons were made by a one-way ANOVA with Dunnett’s multiple comparison test. All data are expressed as mean ± SEM. *p < 0.05, **p < 0.01. **(D)** Graft-bearing EFP recovered at 30 days post-transplantation and stained for GFP (green) and insulin (red). Scale bar = 100 µm. **(E)** Immunohistological staining for GFP (green), CD34 (red) and DAPI (blue). The yellow color represents the GFP-HUVECs with positive staining of anti-CD34. Arrows indicate chimeric blood vessels. Arrowheads indicate red blood cells. Scale bar for top panel = 100 µm and for the 3 bottom panels, 20 µm.

Figure 5 summarizes the results obtained with this group. Blood glucose control was significantly lower in the IC + HUVEC group in comparison to the PIO group (Figure 5A). The IPGTT performed at 30 days post-transplantation demonstrated a poor glucose clearance in the IC + HUVEC group (Figure 5B). Response to increased blood glucose levels was significantly lower than for the PIO group as demonstrated by the AUC (2044 ± 578.1 vs. 966.8 ± 113.7, *p* = 0.008, respectively; Figure 5C).

After demonstrating that incorporation of supportive cells into the PIO improved graft revascularization, we investigated the degree to which these cells were contributing to new vessel development in the graft. To easily identify donor-derived new vessels, GFP-transduced HUVECs were incorporated into the PIO. Graft-bearing EFPs were recovered at 30 days post-transplantation and processed for immunohistological analysis. Interestingly, GFP positive cells were found inside the graft in the PIO group, while none was found in the IC + HUVEC group (Figure 5D). Both human and mouse vessels were positively stained by anti-CD34 confirming the establishment of anastomoses between donor derived HUVECs and mouse blood vessels. Furthermore, GFP/CD34 double positive endothelial cells were found at the graft periphery, inside capillaries containing erythrocytes, indicating that HUVECs were able to migrate and merge with a murine vascular system, forming functionally perfused blood vessels, as shown in Figure 5E. These data indicate that hAECs support HUVECs inside the organoids and thus contribute to accelerated revascularization.

## Discussion

Impaired and delayed revascularization of the graft is a major issue in islet transplantation and represents a main limitation to the search for extrahepatic sites for islet transplantation. Common vascularization strategies focus either on the combination of accessory cells with islets (32) or incorporation of endothelial cells into islet-like constructs generated from embryonic stem cell-derived ß cells (30) or ß cell lines (31), and are mainly based on *in vitro* testing. In this study, we successfully generated functional pre-vascularized islet organoids using multiple cell types. The major finding of this study is that incorporation of hAECs and HUVECs into insulin-producing organoids hastens the rate of graft revascularization, and subsequently results in better engraftment of the β-cell mass.

HUVECs are the most commonly used, robust source of human endothelial cells in regenerative medicine and tissue engineering (33). However, limited proliferative potential of these cells hinders their clinical application. hAECs isolated from the amniotic membrane of discarded placenta is considered a non-controversial stem cell source (34). These cells demonstrated profound anti-fibrotic, anti-inflammatory, non-tumorigenic and low antigenic properties (35, 36). Furthermore, hAECs possess pluripotent stem cells characteristics, can be isolated in large quantities and are thus considered as an evolving therapeutic tool for the development of various clinical applications (35). Previously, we have shown that the generation of insulin-secreting organoids from primary IC in combination with hAECs improved islet engraftment and vascularization primarily by stimulating *VEGF-A* production from the graft *via* HIF1- α signaling pathway (17, 20). Therefore, in this study, we evaluated whether hAECs could accelerate the angiogenic potential of mature endothelial cells (HUVECs). Our results show that chimeric, prevascularized insulin secreting organoids are capable of establishing new vascular networks *in vitro* and *in vivo* when co-cultured with hAECs and HUVECs. The enhancement of the angiogenic potential of HUVECs by hAECs can be explained by three possible mechanisms: 1) *via* the secretion of ECM-degrading proteases facilitating EC migration and sprouting (37), 2) by up-regulating VEGF expression in endothelial and islet cells (38), and 3) by the reduction or suppression of inflammatory responses (39, 40). Our *in vivo* experiments have demonstrated the superiority of pre-vascularized islet organoids for insulin secretion and revascularization.

**FIGURE 6:**
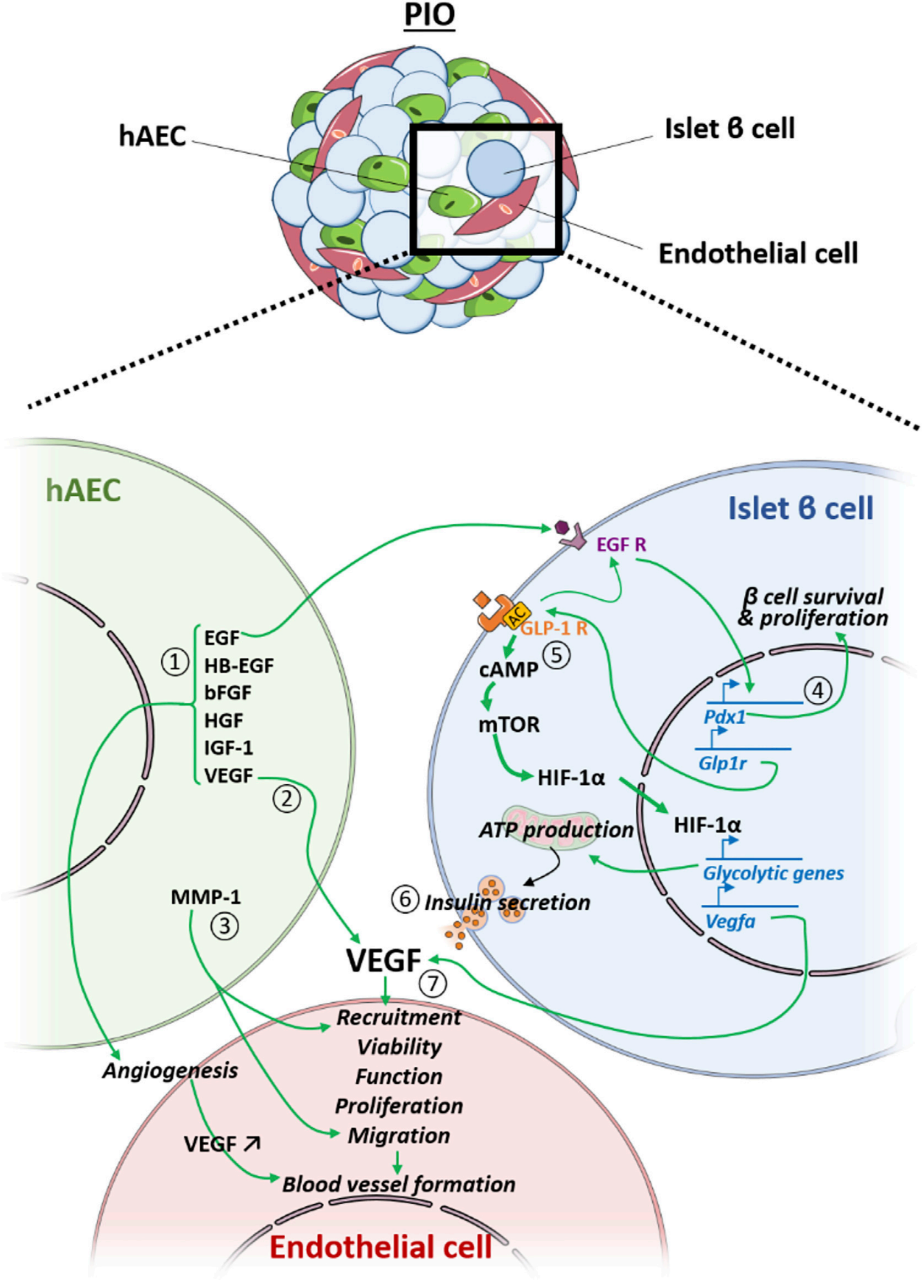
Crosstalk between the hAEC, the endothelial cell (EC) and the islet β cell (IC) within the PIO. hAEC enhances revascularization of the PIO in a direct manner by secreting 1) angiogenic factors and 2) *vegf* that improve EC viability, function, proliferation and blood vessel formation, and 3) by producing ECM-degrading proteases (MMP-1) that facilitate EC migration and sprouting. Additionally, hAECs secrete EGF that 4) upregulates IC *pdx1* expression, leading to higher IC survival and proliferation, as well as 5) *glp1-r* expression, leading to an up-regulation of glycolytic genes and *vegf-a* through the mTOR/HIF-1a pathway, resulting in 6) an improved insulin secretion and 7) a better revascularization of the PIO.

Another important finding is the existence of a cross-talk between the islet, endothelial and amniotic epithelial cells associated within one organoid (summarized in Figure 6), and that this communication can be successfully employed for improving outcomes of islet transplantation. In terms of revascularization, we observe that both blood vessel density and number of functional vessels were significantly higher in the grafts explanted from mice transplanted with PIO in comparison to control groups. *VEGF-A* is a proangiogenic factor that recruits endothelial cells and circulating endothelial progenitors (11). Our results demonstrated significant upregulation of *VEGF-A* gene expression in the grafts explanted from mice transplanted with pre-vascularized organoids. Immunohistochemical analysis of the explanted grafts confirmed that the major producers of *VEGF-A* were islet cells. This finding was in agreement with our previous studies, demonstrating that hAECs markedly increase production of *VEGF-A* in islet cells *via* paracrine signalling (17). In addition, hAECs themselves are known to secrete *VEGF-A* (41), which on the other hand could also enhance performance of HUVECs within the organoids. To verify this hypothesis, we used GFP-HUVECs and tracked transplanted cells inside the graft. We found GFP-HUVECs both inside and in the vicinity of the graft. At the same time, GFP-HUVECs were also detected to be integrated into the peri-islet functional blood vessels containing red blood cells. This indicates that the donor derived endothelial cells anastomosed with the murine vascular system and formed functionally perfused blood vessels. Interestingly, the same was not observed in mice transplanted with IC + HUVECs, in which no GFP-HUVECs were found in the recovered grafts. In addition, almost no blood circulation was observed inside the graft area. This indicates that hAECs contribute to the process of endothelial cell remodelling and stabilization finally leading to mature vessel formation. Our findings are in agreement with previously reported data, demonstrating that hAECs enhance EC viability, function, proliferation, migration and blood vessel formation *in vitro* and *in vivo* (41). Furthermore, amniotic cells secrete additional factors that are critical for angiogenesis, such as EGF, HB-EGF, bFGF, HGF, IGF-1 (42). Taken together, these data suggest that hAECs promote revascularization both directly by secreting angiogenic factors and indirectly by stimulating *VEGF-A* secretion by islet cells.

Accelerated revascularization can also provide important survival cues to the islet cells. Another important challenge to islet transplantation is to achieve stable, long-term insulin independence, preferably with single donor islet transplantation. In this study, improved revascularization was accompanied by prompt return of severely diabetic mice to a normoglycaemic state after transplantation of minimal mass of prevascularized islet organoids. Mice transplanted with PIO showed significantly improved insulin secretion and better glucose clearance compared to mice transplanted with PI, NI and IC + HUVECs. Investigations of underlying mechanisms showed that superior function of β-cells in PIOs was mediated by the GLP-1R signalling pathway. GLP-1R has been found to regulate homeostasis of β-cell mass by inducing β-cell proliferation and protecting against apoptosis. On the other hand, activation of the GLP-1R leads to the activation of multiple downstream pathways, including EGF receptor signalling (43), which in turn stimulates proliferation of β cells (44). EGF has been shown to enhance glucose-dependent insulin secretion and upregulate PDX1 expression (20). Although the precise mechanisms underlying this pattern of increased gene expression in the PIOs are not fully understood, we speculate that growth factor expression profile of hAECs, mainly EGF, could stimulate upregulation of the expression of genes involved in β-cell function (GLP-1R, PDX-1).

## Conclusion

In this study, we demonstrate a novel approach to generate pre-vascularized islet organoids by combining primary ICs with two additional supportive cell types, HUVECs and hAECs, and address some of the challenges of clinical islet transplantation such as donor supply scarcity, impaired islet engraftment and revascularization. Furthermore, our data demonstrate that hAECs not only promote cell viability and engraftment, but most importantly, play a primordial supporting role in the development of HUVEC-derived neo-vessels within the transplanted tissue.

However, to generate large numbers of uniform, size-controlled and functional prevascularized islet organoids, a scalable platform technology is a prerequisite to ensure standardization and reproducibility for new and innovative beta cell replacement strategies.

Addressing this challenge, recently, we showed that several spheroid generating methods are suitable to assemble uniform, size-controlled and functional islet-like clusters (45). The compared techniques included native islets as controls (IEQs), a self-aggregation technique, the hanging drop technique, the agarose 3D microwell technique and the Sphericalplate SP5D. We demonstrated that up to 9000 islet organoids can be easily generated per plate.

Moreover, the SP5D can be automatized, and robotic-mediated spheroid generation can further reduce variability and therefore improve standardization and reproducibility.

Taken together, these findings could be a basis for the design of novel extra-hepatic, extra-vascular islet transplantation sites.

## Capsule Sentence Summary

The pre-vascularized islet organoids were generated from dissociated islet cells, human amniotic epithelial cells (hAECs), and human umbilical vein endothelial cells (HUVECs). Our study demonstrates that pre-vascularized islet organoids exhibit enhanced *in vitro* function and most importantly, improved engraftment and accelerated vascularization *in vivo* in a murine model.

## Vanguard Consortium

The School of Medicine of the Università del Piemonte Orientale “Amedeo Avogadro”: Chiara Borsotti, Simone Merlin. IRCCS Ospedale San Raffaele: Lorenzo Piemonti, Antonio Citro, Silvia Pellegrini. Ludwig-Maximilians-Universität München: Jochen Seissler, Lelia Wolf-van Buerck, Mohsen Honarpisheh. Lyon Claude Bernard University: Olivier Thaunat. Erasmus University Medical Center Rotterdam: Emma Massey, Antonia Cronin, Eline Bunnik, Dide de Jongh. European Society for Organ Transplantation: Luca Segantini, Giovanna Rossi. Kugelmeiers AG: Patrick Kugelmeier, Petra Wolint. Accelopment Switzerland Ltd. : Marco Cavallaro, Julia Götz, Jeanette Müller.

## Data Availability

The original contributions presented in the study are included in the article/Supplementary Material. Further inquiries can be directed to the corresponding author.

## Glossary

AMCA: Aminomethylcoumarin Acetate
AUC: area under the curve
bFGF: basic fibroblast growth factor
BSA: Bovine Serum Albumine
CCER: Commission Cantonale d’Ethique de la Recherche
CK-7: Cytokeratin 7
DAPI: 4′,6-diamidino-2-phénylindole
DMEM: Dulbecco’s Modified Eagle Medium
EC: endothelial cell
EFP: epididymal fat pad
EGF: epidermal growth factor
FBS: fetal bovine serum
GFP: green fluorescent protein
GLP-1R: Glucoagon-like peptide 1 receptor
hAEC: human amniotic epithelial cell
HB-EGF: heparin binding epithelial growth factor
HBSS: Hanks’ balanced salt solution
HGF: Hepatocyte growth factor
HIF1-a: Hypoxia-inducible factor 1-alpha
HUVEC: human umbilical vein endothelial cell
IC: islet cell
IEQ: islet equivalent
IGF-1: insulin-like growth factor-1
IPGTT: intraperitoneal glucose tolerance test
LV: lentiviral
MEM-NEAA: Minimum essential medium non-essential amino acids
MOI: multiplicity of infection
NI: native islet
NDC: non-diabetic control
PBS: Dubbelco’s Phosphate buffer saline
PCSK1: Proprotein Convertase Subtilisin/Kexin Type 1
PCSK2: Proprotein Convertase Subtilisin/Kexin Type 2
PDX-1: pancreatic and duodenal homeobox 1
PFA: Paraformaldehyde
PI: pseudo-islet
PIO: prevascularized islet organoid
RPLP1: ribosomal protein lateral stalk subunit P1
RT-PCR: reverse transcriptase polymerase chain reaction
SI: stimulation index
STZ: streptozotocin
VEC: vascular endothelial cadherin
VEGF-A: Vascular endothelial growth factor A
