# Supplementary material for: Bio-Engineering of Pre-Vascularized Islet Organoids for the Treatment of Type 1 Diabetes
Source: Transpl Int. 2022 Jan 21;35:10214. doi: 10.3389/ti.2021.10214 (PMC8842259; doi:10.3389/ti.2021.10214)
Supplement: Supplementary file 1 [file DataSheet2.pdf]

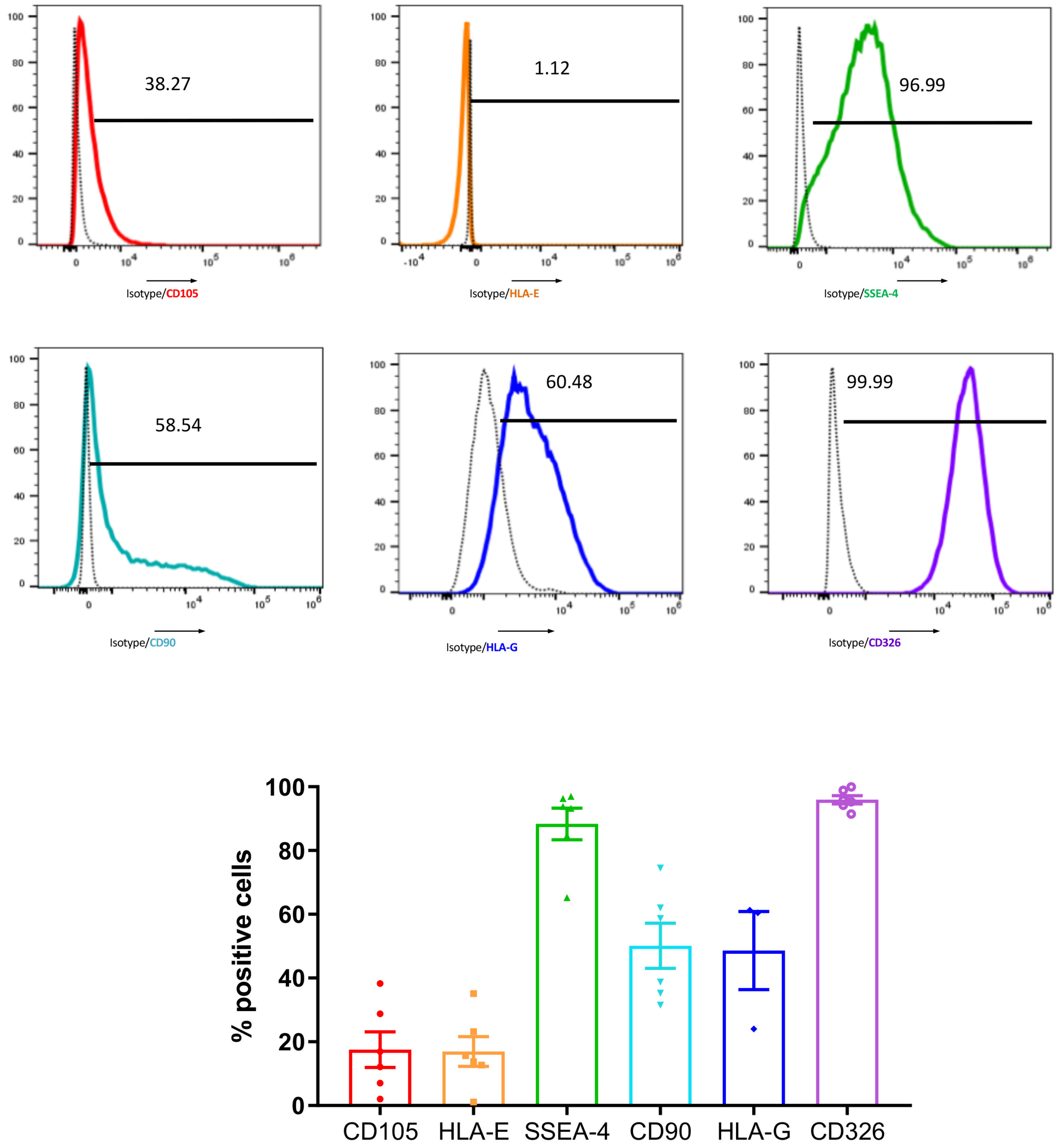

Figure S1. Flow cytometry analysis of the hAECs derived from 6 different placentae. Results are expressed as the percentage of positive cells for the following markers: CD105, HLA-E, SSEA-4, CD90, HLA-G and CD326. Three data are missing for HLA-G analysis.

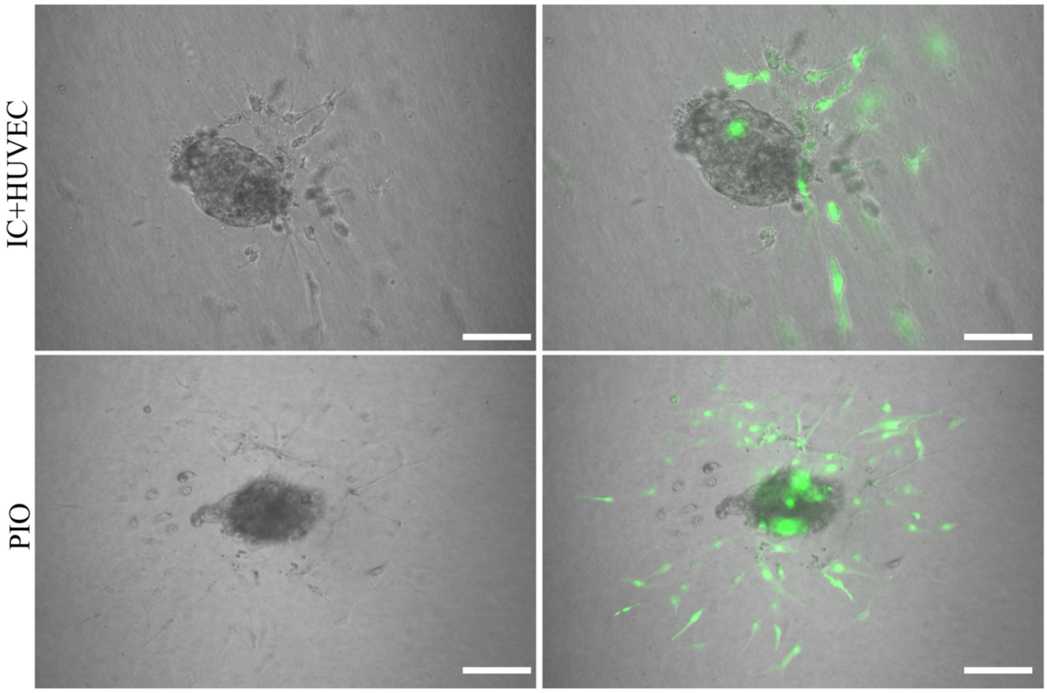

Figure S2. IC+HUVEC and PIO 3D sprouting assay. One hundred spheroids composed of IC + GFP-transduced HUVEC and 100 PIO were recovered after 4 days in culture and placed for 24 hours at 37°C in 5mg/ml collagen gel, supplemented with 200ng/ml of VEGF. Light microscope pictures of IC+HUVEC spheroids and PIO were taken inside the hydrogel. GFP-positive cells are spontaneously green. Scale bar = 75 µm.

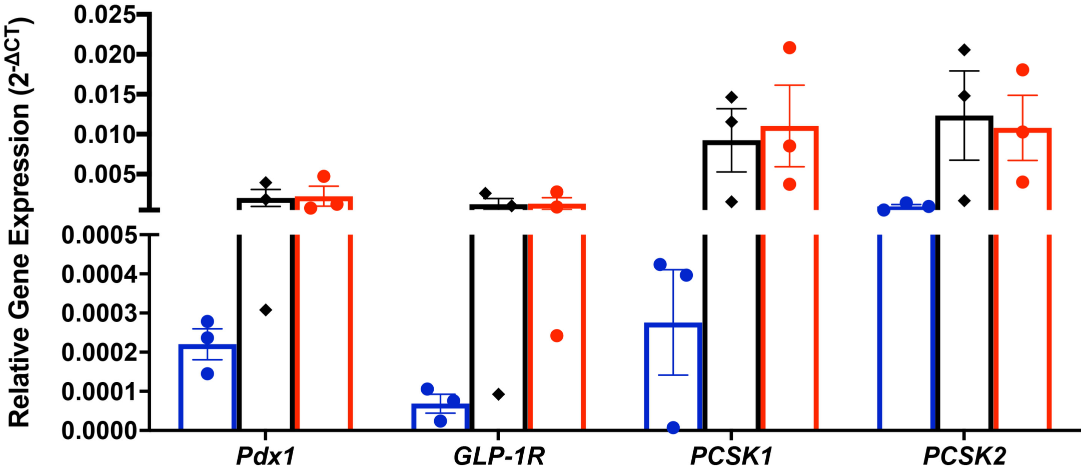

Figure S3. Gene expression by qPCR at 30-days post-transplantation of the following genes: *pdx1* (pancreatic and duodenal homeobox 1), *glp-1r* (glucagon-like peptide-1 receptor), *pcsk1* (proprotein convertase 1) and *pcsk2* (proprotein convertase 2) in PIO, PI and NI (n=3 mice in each group). All data are expressed as mean ± SEM.
