# Supplementary material for: Bio-Engineering of Pre-Vascularized Islet Organoids for the Treatment of Type 1 Diabetes
Source: Transpl Int. 2022 Jan 21;35:10214. doi: 10.3389/ti.2021.10214 (PMC8842259; doi:10.3389/ti.2021.10214)
Supplement: Supplementary file 2 [file DataSheet1.docx]

| **Supplementary table 1.** Primary and secondary antibodies | | | | | | | |
| --- | --- | --- | --- | --- | --- | --- | --- |
|  | **Antibody** | **Company** | **City** | **Country** | **Product number** | **Application** | **Dilution** |
| **Primary antibody** | PerCP-Cy 5.5 Mouse anti-SSEA-4 | BD Biosciences | Allschwil | Switzerland | 561565 | FC | 1:50 |
|  | PE-Cy 7 Mouse anti-human CD90 | BD Biosciences | Allschwil | Switzerland | 561558 | FC | 1:100 |
|  | FITC Mouse anti-human CD105 | BD Biosciences | Allschwil | Switzerland | 561443 | FC | 1:50 |
|  | BV421 Mouse anti-human CD326 | BD Biosciences | Allschwil | Switzerland | 563180 | FC | 1:50 |
|  | PE anti-human HLA-E | Biolegend | London | UK | 342604 | FC | 1:50 |
|  | APC anti-human HLA-G | Biolegend | London | UK | 335910 | FC | 1:50 |
|  | PE anti-human CD31 | Biolegend | London | UK | 303106 | FC | 1:30 |
|  | AlexaFluor 657 CD144 | BD Biosciences | Allschwil | Switzerland | 561567 | FC | 1:40 |
|  | PerCP-Cy 5.5 anti-human CD45 | Biolegend | London | UK | 368504 | FC | 1:25 |
|  | PerCP-Cy 5.5 mouse IgG1, κ Isotype Ctrl | Biolegend | London | UK | 400150 | FC | isotype 1:60 |
|  | PE mouse IgG1, κ Isotype Ctrl | Biolegend | London | UK | 400112 | FC | isotype 1:40 |
|  | AlexaFluor 657 mouse IgG1, κ Isotype Ctrl | BD Biosciences | Allschwil | Switzerland | 557714 | FC | isotype 1:100 |
|  | PerCP-Cy 5.5 Mouse IgG3, κ Isotype Ctrl | BD Biosciences | Allschwil | Switzerland | 561572 | FC | 1:50 |
|  | PE-Cy 7 Mouse IgG1, κ Isotype Ctrl | BD Biosciences | Allschwil | Switzerland | 557872 | FC | 1:100 |
|  | FITC Mouse IgG1, κ Isotype Ctrl | BD Biosciences | Allschwil | Switzerland | 555748 | FC | 1:50 |
|  | BV421 Mouse IgG1, κ Isotype Ctrl | BD Biosciences | Allschwil | Switzerland | 562438 | FC | 1:50 |
|  | PE Mouse IgG1, κ Isotype Ctrl | Biolegend | London | UK | 400112 | FC | 1:50 |
|  | APC Mouse IgG2a, κ Isotype Ctrl | Biolegend | London | UK | 400222 | FC | 1:50 |
|  | Purified rabbit anti-human CD34 | Abcam | Cambridge | UK | ab81289 | FC, IF | 1:50 (FC), 1:2000 (IF) |
|  | Rabbit polyclonal anti wide spectrum Cytokeratin | Abcam | Cambridge | UK | ab9377 | IF | 1:75 |
|  | GuineaPig anti-insulin | DakoCytomation | Baar | Switzerland | A0564 | IF | 1:100 |
|  | Rabbit anti-human VEGF | Santa-Cruz biotechnology | Dallas | USA | SC-152 | IF | 1:100 |
|  | Mouse anti-vimentin | DakoCytomation | Baar | Switzerland | M0725 | IF | 1:50 |
|  | Mouse anti-human CD31 | DakoCytomation | Baar | Switzerland | M0823 | IF | 1;50 |
|  | Rabbit anti human Von Wilderbrand Factor | DakoCytomation | Baar | Switzerland | A0082 | IF | 1:100 |
|  | Chicken anti-GFP | Abcam | Cambridge | UK | ab13970 | IF | 1;500 |
|  | Rabbit anti-GFP | Abcam | Cambridge | UK | ab6556 | IF | 1;500 |
| **Secondary antibody** | AlexaFluor 488 goat anti-mouse IgG | ThermoFisher Scientific | Reinach | Switzerland | A11001 | IF | 1:300 |
|  | AlexaFluor 488 goat anti-chicken IgG | Abcam | Cambridge | UK | ab150173 | IF | 1;500 |
|  | AlexaFluor 488 goat anti-rabbit IgG | ThermoFisher Scientific | Reinach | Switzerland | A11008 | IF | 1:300 |
|  | AlexaFluor 555 goat anti-rat IgG | ThermoFisher Scientific | Reinach | Switzerland | A21434 | IF | 1:300 |
|  | AlexaFluor 555 donkey anti-rabbit IgG | ThermoFisher Scientific | Reinach | Switzerland | A31572 | IF | 1:300 |
|  | AlexaFluor 555 goat anti-guinea pig IgG | ThermoFisher Scientific | Reinach | Switzerland | A21435 | IF | 1:300 |
|  | AlexaFluor 555 donkey anti-mouse IgG | ThermoFisher Scientific | Reinach | Switzerland | A31570 | IF | 1:300 |
|  | Donkey anti-guinea pig Fluorescein IgG | Jackson ImmunoResearch Laboratories | Rheinfelden | Switzerland | 706-095-148 | IF | 1:200 |
|  | Donkey anti Mouse Rhodamine IgG | Jackson ImmunoResearch Laboratories | Rheinfelden | Switzerland | 715-025-150 | IF | 1:200 |
|  | Goat anti GuineaPig Fluorescein IgG | Jackson ImmunoResearch Laboratories | Rheinfelden | Switzerland | 106-095-003 | IF | 1:200 |

FC: Flow cytometry, IF: immunofluorescence, UK: United Kingdom

| **Supplementary table 2.** Culture medium, reagents and materials | | | | | | | | |
| --- | --- | --- | --- | --- | --- | --- | --- | --- |
|  | **Reagents** | **Company** | **City** | **Country** | **Product number** | **Concentration** | **Application** | |
| **HUVEC culture medium** | Medium 199 | ThermoFisher Scientific | Reinach | Switzerland | 21180021 | 1X | |  |
|  | Fetal bovine serum (FBS) | Merk Millipore | Zug | Switzerland | s0115 | 10% v/v | |  |
|  | L-Glutamin-Penicillin-Streptomycin 10X | Sigma Aldrich | Buchs | Switzerland | G1146 | Details of the product in the legend^§^ | |  |
|  | fungin | Invivogen | San Diego | USA | ant-fn-2 | 0.1% v/v | |  |
|  | Endothelial cell growth supplement (ECGs) | Sigma Aldrich | Buchs | Switzerland | E2759 | 30ug/ml | |  |
|  | Heparin |  |  |  |  | 100ug/ml | |  |
| **hAEC culture medium** | Fetal bovine serum (FBS) | Merk Millipore | Zug | Switzerland | s0115 | 10% v/v | |  |
|  | Sodium Pyruvate | Sigma Aldrich | Buchs | Switzerland | s8636 | 1 mmol/l | |  |
|  | L-Glutamin-Penicillin-Streptomycin 10X | Sigma Aldrich | Buchs | Switzerland | G1146 | Details of the product in the legend^§^ | |  |
|  | DMEM/F-12 | ThermoFisher Scientific | Reinach | Switzerland | 21041-25 | 1X | |  |
|  | MEM NEAA 100X | ThermoFisher Scientific | Reinach | Switzerland | 11140-035 | 1% v/v | |  |
|  | fungin | Invivogen | San Diego | USA | ant-fn-2 | 0.1% v/v | |  |
|  | 2-mercaptoethanol | ThermoFisher Scientific | Reinach | Switzerland | 21985-023 | 1 mmol/l | |  |
|  | human recombinant epidermal growth factor (hEGF) | Sigma Aldrich | Buchs | Switzerland | E9644 | 10 ng/ml | |  |
| **Rat islet culture medium** | DMEM medium | ThermoFisher Scientific | Reinach | Switzerland | 11966025 | 1X | |  |
|  | Fetal bovine serum (FBS) | Merk Millipore | Zug | Switzerland | s0115 | 10% v/v | |  |
|  | Sodium Pyruvate | Sigma Aldrich | Buchs | Switzerland | s8636 | 1 mmol/l | |  |
|  | Glucose 40% | Bichsel | Interlaken | Switzerland | 32 923 373 | 11 mmol/l | |  |
|  | L-Glutamin-Penicillin-Streptomycin 10X | Sigma Aldrich | Buchs | Switzerland | G1146 | Details of the product in the legend^§^ | |  |
| **Reagents and materials for isolation / dissociation of islets and cells and spheroid characterization** | Collagenase A | Sigma Aldrich | Buchs | Switzerland | 10103578001 | 2mg/mL | | HUVEC isolation |
|  | Collagenase V | Sigma Aldrich | Buchs | Switzerland | C9263-5G | 1mg/mL | | Rat islet isolation |
|  | 0.05% (w/v) trypsin-EDTA | ThermoFisher Scientific | Reinach | Switzerland | 25300-054 | - | | hAEC isolation/ rat islet dissociation |
|  | HBSS | ThermoFisher Scientific | Reinach | Switzerland | 14175-053 | - | | hAEC isolation |
|  | Antibiotic/antimycotic solution | Sigma Aldrich | Buchs | Switzerland | A5955 | 100 U/ml penicillin, 100 mg/ml streptomycin and 0.25 mg/ml amphotericin B | | hAEC isolation |
|  | Dulbecco's Phosphate Buffer Saline (PBS) | Sigma Aldrich | Buchs | Switzerland | D8537 | - | | FC |
|  | Bovine Serum Albumine (BSA) | Sigma Aldrich | Buchs | Switzerland | A3733 | 0.1% w/v | | FC |
|  | NaCl 0.9% | Bichsel | Interlaken | Switzerland | 100 0 178 | - | | Spheroid generation |
|  | CM-DiL | ThermoFisher Scientific | Reinach | Switzerland | C7000 | 0,736111111 | | Spheroid generation |
|  | Streptozotocin | Sigma Aldrich | Buchs | Switzerland | s0130 | 12.6 mg/mL | | In vivo diabetes induction |
|  | Paraformaldehyde (PFA) | Sigma Aldrich | Buchs | Switzerland | P6148-1KG | 4% w/v | | IF |
|  | DAPI ProTaqs MountFluor Anti-Fading | Quartett Biochemicals | Berlin | Germany | 401603392 | - | | IF |
|  | Matrigel Matrix | Corning | New-York | USA | 356234 | - | | HUVEC in vitro assessment |
|  | VEGF | ThermoFisher Scientific | Reinach | Switzerland | PHC9393 | 200ng/ml | | - |
|  | DyLight 649 Griffonia Simplicifolia Lectin-Isolectin B4 | Reactolab | Servion | Switzerland | DL-1208-.5 | undiluted | | IF |

§ : 1% v/v (2 mmol/l L-Glutamin, 100 U/ml Penicillin, 0.1 mg/ml (mmol/l L-Glutamin, 100 U/ml Penicillin, 0.1 mg/ml Streptomycin)

| **Supplementary table 3.** Kits, instruments and softwares | | | | | | |
| --- | --- | --- | --- | --- | --- | --- |
|  | **Materials** | **Company** | **City** | **Country** | **Product number** | **Application** |
| **RT qPCR** | Rneasy minikit | Qiagen | Courtaboeuf | France | 74104 |  |
|  | High Capacity cDNA Reverse transcription kit | ThermoFisher Scientific | Reinach | Switzerland | 4368814 |  |
|  | TaqMan Fast Advance Master Mix | ThermoFisher Scientific | Reinach | Switzerland | 4444557 |  |
|  | Rat insulin ELISA kit | Mercodia | Uppsala | Sweden | 10-1250-01 |  |
| **Instruments/Software** | Galios cytometer | Beckman Coulter | Indianapolis | Indiania (USA) | - | Flow cytometry |
|  | Kaluza Analysis software (version 1.5.20365.16139) | Beckman Coulter | Indianapolis | Indiania (USA) | - | Flow cytometry |
|  | DMi8 manual microscope | Leica Microsystems | Heerbrugg | Switzerland |  | - |
|  | Nikon A1R | Nikon Imaging | Egg | Switzerland | - | IF |
|  | NIS‐Elements Imaging Software (version 4.20.00 Build 972) | Nikon Imaging | Egg | Switzerland | - | IF |
|  | Zeiss Axiocam | Zeiss | Feldbach | Germany | - | IF |
|  | Zeiss Axioscan.Z1 slide scanner | Zeiss | Feldbach | Germany | - | IF |
|  | Zen 2.3 Blue Edition software (version 2.3.60.1000) | Zeiss | Feldbach | Germany | - | IF |
|  | ImageJ software | NIH | Bethesda | Maryland (USA) | - | - |
|  | AggreWellTM400 24-well plates | Stemcell Technologies | Köln | Germany | [34415](https://www.sigmaaldrich.com/catalog/product/sigma/z764000?lang=fr&region=CH) | Spheroid generation |
|  | Ibidi microscopy culture chambers | Ibidi | Planegg | Germany | 81158 | IF |
|  | Freestyle Precision glucometer | Abbott Diabetes Care | Baar | Switzerland | - | in vivo |
|  | PE50 tubing | PhyMep | Paris | France | BTPE-50 | in vivo transplantation |
|  | Screw-drive syringe | Hamilton | Reno | Nevada (USA) | 81341 | in vivo transplantation |
|  | Prism software 8.0 | GraphPad | La Jolla | California (USA) | - | Statistics |

**Table 4.** Rat primers used for gene amplification

| **Gene** | **Forward sequence** | **Reverse sequence** |
| --- | --- | --- |
| *RPLP1* | TCT CTG AGC TTG CCT GCA TCT ACT | CCT ACA TTG CAG ATG AGG CTT CCA |
| *INS* | AGC AAG CAG GTC ATT GTT CC | ACC AGG TGA GGA CCA CAA AG |
| *Pdx1* | TGC CAC CAT GAA TAG TGA GG | CAG GGG GAT TAG CAC TGA AC |
| *GLP-1R* | TGG GGG AGA TAC AAC AGA GG | CTC TGG GCT TCT CAA CTT GG |
| *PCSK1* | GCA AAG AGG TTG GAC TCT GC | TCT GGC CCT CCA TGT ATC TC |
| *PCSK2* | TGT CTC TGC CTC TCC TTG GT | TGA GAG CAA GCA AAG CTT CA |
| *VEGF-A* | GGT AAT GGC TCC TCC TCC TC | AAG CCA CTC ACA CAC ACA GC |
